# Does the Score on the MRC Strength Scale Reflect Instrumented Measures of Maximal Torque and Muscle Activity in Post-Stroke Survivors?

**DOI:** 10.3390/s21248175

**Published:** 2021-12-07

**Authors:** Pawel Kiper, Daniele Rimini, Deborah Falla, Alfonc Baba, Sebastian Rutkowski, Lorenza Maistrello, Andrea Turolla

**Affiliations:** 1 Physical Medicine and Rehabilitation Unit, Azienda ULSS 3 Serenissima, 30126 Venice, Italy; 2 Medical Physics Department-Clinical Engineering, Salford Care Organisation, Salford M6 8HD, UK; daniele.rimini@srft.nhs.uk; 3 Centre of Precision Rehabilitation for Spinal Pain (CPR Spine), School of Sport, Exercise and Rehabilitation Sciences, University of Birmingham, Birmingham B15 2TT, UK; d.falla@bham.ac.uk; 4 Rehabilitation Unit, Azienda Ospedale Università Padova, 35128 Padua, Italy; alfonc.baba@aopd.veneto.it; 5 Faculty of Physical Education and Physiotherapy, Opole University of Technology, 45-758 Opole, Poland; s.rutkowski@po.opole.pl; 6 Laboratory of Neurorehabilitation Technologies, San Camillo IRCCS, 30126 Venice, Italy; lorenza.maistrello@hsancamillo.it

**Keywords:** stroke, neurorehabilitation, EMG, MRC, dynamometer, strength

## Abstract

It remains unknown whether variation of scores on the Medical Research Council (MRC) scale for muscle strength is associated with operator-independent techniques: dynamometry and surface electromyography (sEMG). This study aimed to evaluate whether the scores of the MRC strength scale are associated with instrumented measures of torque and muscle activity in post-stroke survivors with severe hemiparesis both before and after an intervention. Patients affected by a first ischemic or hemorrhagic stroke within 6 months before enrollment and with complete paresis were included in the study. The pre- and post-treatment assessments included the MRC strength scale, sEMG, and dynamometry assessment of the triceps brachii (TB) and biceps brachii (BB) as measures of maximal elbow extension and flexion torque, respectively. Proprioceptive-based training was used as a treatment model, which consisted of multidirectional exercises with verbal feedback. Each treatment session lasted 1 h/day, 5 days a week for a total 15 sessions. Nineteen individuals with stroke participated in the study. A significant correlation between outcome measures for the BB (MRC and sEMG *p* = 0.0177, ρ = 0.601; MRC and torque *p* = 0.0001, ρ = 0.867) and TB (MRC and sEMG *p* = 0.0026, ρ = 0.717; MRC and torque *p* = 0.0001, ρ = 0.873) were observed post intervention. Regression models revealed a relationship between the MRC score and sEMG and torque measures for both the TB and BB. The results confirmed that variation on the MRC strength scale is associated with variation in sEMG and torque measures, especially post intervention. The regression model showed a causal relationship between MRC scale scores, sEMG, and torque assessments.

## 1. Introduction

The primary aim of the post-stroke rehabilitation process is to restore and maintain the patient’s ability to perform actives of daily living. Importantly, this process starts within the first days after stroke and often continues over many years [1]. Because one of the most evident consequences of a cerebrovascular injury is hemiparesis, the rehabilitation process requires accurate assessment of residual muscle activity to define rehabilitative requirements [2,3]. Hemiparesis is associated with muscle weaknesses and inability to produce adequate muscle force for task execution. The ability to generate muscle force is determined by neural, muscular, and biomechanical factors. The contraction that is generated following depolarization of the nervous cells, which release calcium ions, giving start to the process of contraction in the main body of the muscle fiber [4], can be evaluated either in terms of muscular activity or the resultant muscular force.

The status or change of a clinical condition is traditionally assessed by completing assessment scales. The validity and reliability of these assessment scales are essential to monitor a patient’s recovery and are critical for determining appropriate therapies. A common and widely accepted assessment scale for muscle strength is the Medical Research Council (MRC) scale [5,6]. This scale is commonly used to evaluate patients with stroke suffering from muscle weakness due to hemiplegia [7]. The MRC scale for muscle strength uses manual muscle testing to grade muscle strength, ranging from 0 to 5, according to the maximum force expected for a certain muscle [6]. The grades are as follows: 0 = No contraction, 1 = Flicker or trace contraction, 2 = Active movement, with gravity eliminated, 3 = Active movement against gravity, 4 = Active movement against gravity and resistance, and 5 = Normal power. A modified version of the scale takes into account the evaluation of range of movement (ROM), and the grades are as follows: 0 = No contraction, 1 = Flicker or trace contraction, 2 = Active movement, with gravity eliminated, 2–3 = Active movement against gravity over less than 50% of the feasible ROM, 3 = Active movement against gravity over more than 50% of the feasible ROM, 3–4 = Active movement against resistance over less than 50% of the feasible ROM, 4 = Active movement against resistance over more than 50% of the feasible ROM, 4–5 = Active movement against strong resistance over the feasible ROM, but distinctly weaker than the contralateral side, 5 = Normal power. The reliability of the MRC scale and its modified versions (mMRC) have been investigated [8]. Substantial inter-rater reliability of the MRC scale and the mMRC scale as well as intra-rater reliability of the MRC and the mMRC was observed for forearm muscle evaluation [8]. Additionally, the validity of the MRC scale has been confirmed, thus supporting its use in clinical practice. Jepsen et al. examined the inter-rater reliability of manual muscle tests of maximal voluntary strength and observed that reduced strength was significantly associated with the presence of symptoms; they suggested that manual muscle testing in upper limb disorders has diagnostic potential [9]. However, some limitations exist relating to the interpretation of the MRC scale, for example, the width of the MRC grades are unequal [10]. For instance, when testing elbow flexion strength, the MRC grades overlap between grades 3 and 4, indicating that the MRC grading may be unreliable in quantifying elbow flexion strength. Furthermore, excluding patients with either grade 0 or grade 5 decreases the reliability of the MRC Scale [8]. This may indicate that the assessment of the different grades of impairment can be more difficult than the assessment of a muscle that has no contraction at all or is evaluated with its maximal strength. An additional consideration is that Dupépé et al. suggested that the inter-observer reliability of the mMRC scale has discrepancy among trained observers. Additionally, the reliability of the MRC scale varies depending on whether lower-extremity or upper-extremity muscle groups are tested [11,12]. Importantly, however, it remains unknown whether variation of MRC scale scores is associated with a similar variation obtained with operator-independent techniques such as strength measures obtained with dynamometry and quantitative measures of muscle activity such as surface electromyography (sEMG).

Muscle force can be directly measured with a dynamometer, an electromechanical device that can be used to measure muscular strength of a maximum isometric contraction for most major joints in the human body [13,14]. Baschung Pfister et al. evaluated the reliability and validity of manual muscle testing and hand-held dynamometry (HHD) by measuring maximum isometric strength in eight muscle groups across three measurement points. The correlation between the total score on manual muscle testing and HHD was not satisfactory and raised doubt as to whether manual muscle testing measures the same construct (i.e., isometric strength) as HHD [15]. The total score from manual muscle testing was considered reliable and a time-efficient assessment to consider for the detection of general muscle weakness but not for single muscle groups. On the contrary, HHD could be recommended to evaluate isometric muscle strength of single muscle groups [15]. A study by Aguiar et al. revealed that dynamometry provided adequate inter- and intra-rater reliability when used in the subacute phase of stroke [16]. Additionally, recent studies evidenced the utility and the reliability of dynamometry to evaluate force of the paretic side of post-stroke patients [17]. The available literature reports that HHD is an efficient, objective, sensitive, and affordable alternative for strength quantitation [18].

The sEMG is a non-invasive technique for recording the electrical signal generated by muscular activity [19]. Decoding and extracting information contained in this signal provides information on neuromuscular function, which is not provided by other assessment techniques in neurorehabilitation [20]. This data can enhance the characterization of neuromuscular impairments, while tracking the changes in muscle activity from baseline when neurorehabilitation interventions are administered. Clinically, sEMG is frequently used to obtain a precise and objective measure of muscle activity during motor performance [21,22,23,24,25]. EMG is useful to assess hyperactivity and inactivity of selected muscles [26] and, given that it can be used to evaluate the integrity of neuromuscular system, it is often adopted as a physiological biofeedback in physical therapy [27]. In recent decades, the limitations of analyzing EMG have emerged, including physiologically confounding factors [28]. For this reason, pattern recognition techniques have been widely adopted to classify hand gesture [29,30,31], gait analysis [32], and upper limb prosthesis control [33,34,35]. The importance of integrating kinematics and kinetics has also been highlighted [36]. The generation of muscular force assessed by the MRC scale has been associated with the electrical signal observed via sEMG recordings [37]. Furthermore, some mathematical models of motor unit with a parameterization of the electrical and mechanical components of the model were proposed. These models can highlight a physiologically meaningful EMG–force relation in the simulated signals [38]. However, the relationship between muscular force and sEMG during voluntary contractions in pathological conditions (e.g., central nervous system injury) is still poorly understood [39,40].

Thus, in this study we examined whether the scores of the MRC scale are associated with instrumented measures of muscular force and muscle activity pre- and post- an intervention for severe hemiparesis in post-stroke survivors.

Proprioceptive-based training (PBT) was used as a treatment model; PBT is a neuromodulatory treatment modality that has been proposed for the treatment of the upper limb to recover voluntary muscle contraction and strength in stroke survivors [41].

## 2. Materials and Methods

### 2.1. Setting and Participants

This study was conducted in the neurorehabilitation hospital and research institute of San Camillo IRCCS (Venice, Italy). Inpatients affected by first ischemic or hemorrhagic stroke within 6 months before enrollment in the study and with an MRC score at baseline between 0 and 1 point for their biceps brachii and triceps brachii were included in this study. The presence of hypertonia, apraxia, global sensory aphasia, neglect, cognitive impairments, severe sensitivity disorders, stroke lesion located in the cerebellum, or refusal to participate resulted in exclusion from the study.

The local Ethics Committee of the IRCCS San Camillo Hospital approved this study (Protocollo 2012.07 BAT v.1.2), which was registered on ClinicalTrials.gov (NCT03155399). Informed, written consent was obtained prior to participation in the study.

### 2.2. Outcome Measures

The MRC scale for muscle strength, dynamometry measures of maximal elbow flexion and extension torque, and sEMG measures of biceps brachii and triceps brachii activity were implemented pre- and post-intervention. The positions of the upper extremity for dynamometry measurements and for the MRC scale assessment were the same. An elbow splint was used to standardize the position of the patient’s arm during sEMG signal acquisition; the elbow joint was fixed to 40° for assessment of the biceps brachii and 90° for the triceps brachii.

#### 2.2.1. MRC Scale for Muscle Strength

**Figure 1:**
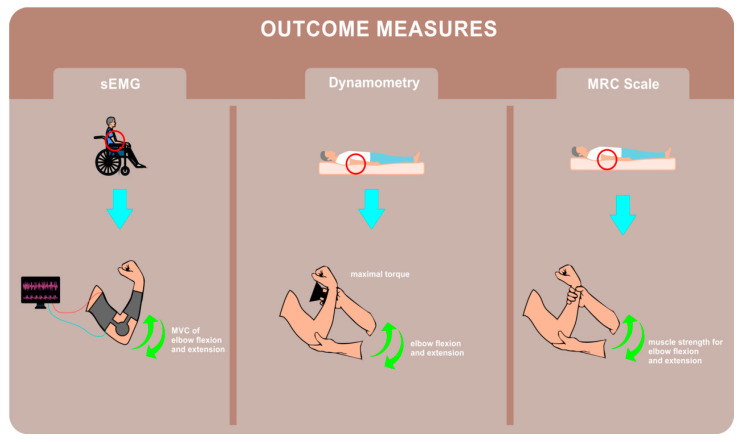
Visualization of the outcome measures applied. sEMG = surface electromyography. MVC = maximal voluntary contraction. MRC Scale = Medical Research Council Scale.

Testing was performed by a physiotherapist after assessment of elbow range of motion. The physiotherapist ensured that the wrist flexors were not contracted when assessing biceps brachii and provided stabilization support with a hand placed above the patient’s elbow when assessing triceps brachii. All patients firstly underwent an assessment of the biceps brachii followed by assessment of the triceps brachii. The assessment of biceps brachii was performed in the supine (or in sitting in the case of grade 2 or more) position with the forearm supinated and elbow flexed to approximately 45 degrees as the patient was asked to “bend your elbow” (Figure 1). The assessment of triceps brachii was performed in the sitting (or in prone in the case of grade 3 or more) position with the arm supported at 90 degrees of shoulder and elbow flexion [5,42] as the patient was asked to “straighten your arm”. For both assessments, the patients performed three attempts and the best result was considered the outcome.

#### 2.2.2. Dynamometry

An electrical dynamometer (CITEC Hand-Held Dynamometer) was used for testing. The participant’s positions for assessment of maximal elbow flexion and extension torque were adopted from the MRC scale evaluation. The biceps brachii was assessed first in all participants. Patients were asked to perform three attempts with verbal encouragement to exceed the previous score and the mean value was considered for analysis.

#### 2.2.3. Surface Electromyography

The sEMG was acquired with bipolar electrodes from the long head of the biceps brachii and the lateral head of the triceps brachii, according to published guidelines for electrode placement [43] after skin preparation. The sEMG signal was amplified with a gain of 1000, band-pass filtered (fifth-order Butterworth filter, bandwidth 10–500 Hz), and sampled at 2048 Hz using a multichannel EMG amplifier (EMG-USB2+ OT Bioelettronica SRL, Torino, Italy). The reference electrode was placed around the wrist of the tested arm. Each linear envelope of EMG activity was obtained by full-wave rectifying and then low-pass filtering (Fc = 6 Hz) for each sEMG channel.

The sEMG was acquired during maximal voluntary isometric contraction (MVC) of elbow flexion and extension, each repeated three times. The sEMG was recorded with the following procedure: recording of baseline activity at a resting state followed by the task itself (i.e., elbow flexion or extension MVC) recorded for 2 s each. The peak values of the amplitude of the envelopes of the sEMG of baseline and during the MVC were extracted, and the difference was computed. The mean value from the three repetitions was considered for further analysis.

### 2.3. Intervention

Participants underwent PBT, which consisted of multidirectional exercises executed synchronously with the unaffected limb and verbal feedback. Patients were asked to move both upper limbs synchronously performing bilateral flexion-extension at the level of their elbow joint. The PBT therapeutic session was divided into the following repetitive phases: proprioceptive stimulation for 3 min with a rest of at least 2 min between stimulations and repeated at least three times for each muscle. Additionally, all participants received individual exercises (passive, active-assisted, or active) for postural control in sitting or standing position. The training protocol lasted 1 h a day, 5 days weekly for a total of 15 sessions [41].

### 2.4. Statistical Analysis

Data distribution for all the variables was tested through the Shapiro–Wilk test. The Spearman’s rank correlation test was used to study potential associations between the MRC scale score and measures of elbow flexion and extension strength and sEMG amplitude of the biceps brachii and triceps brachii muscles both pre- and post-intervention and on the change scores (before–after intervention). A regression model was implemented on the post intervention data to verify the relationship between the MRC strength scale scores and dynamometry measurements of elbow flexion and extension strength and sEMG amplitude of the biceps brachii and triceps brachii. We assessed the MRC models fitting as follows: the overall significance of the regression model with the percentage of variance explained (% Variance explained); the variance of the residuals (Residuals vs. Fitted plot); the normality of the residual distribution (Shapiro–Wilk normality test and Normal QQ-Plot); the presence of outliers (Residuals vs. Leverage plot). Bland–Altman graphs were reported to evaluate the agreement between the measurements made with MRC and those made with sEMG and dynamometry. The statistical significance level was set at *p* < 0.05. All calculations were performed using *R* Statistical Computing software.

## 3. Results

Data from 19 patients with a mean age of 61.48 ± 12.77 years (10 female and 9 male) were analyzed in this study. Patients’ mean time from stroke onset was 3.19 ± 1.80 months. Twelve patients had ischemic stroke and seven had a hemorrhagic stroke (8 right and 11 left lesion side). Descriptive characteristics of the parameters measured before and after intervention are presented in Table 1.

A statistically significant relationship between the outcome measurements was observed pre-intervention between the MRC scale score and dynamometry measures (biceps brachii *p* = 0.0000; triceps brachii *p* = 0.0002) (Table 2), whereas, post-intervention, the MRC scale score was significantly associated with measures of sEMG and dynamometry measures for both biceps brachii (i.e., MRC and sEMG *p* = 0.0177; MRC and Dynamometry *p* = 0.0001) and triceps brachii (i.e., MRC and sEMG *p* = 0.0026; MRC and Dynamometry *p* = 0.0001) (Table 2).

**Figure 2:**
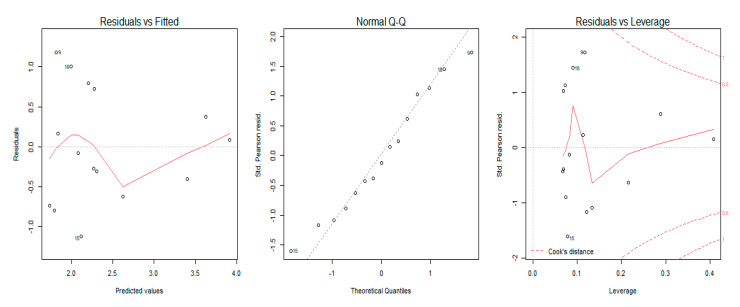
Graphical residual analysis for the MRC model and sEMG for the biceps brachii muscle. The plot on the left shows the residual errors versus their estimated values and the points on the graph should be arranged randomly. The QQ plot in the center shows the distributive normality of the residuals and the points on the plot should follow the diagonal line. The plot on the right identifies any influential data points on the model. In the plot, the Leverage’s values of the points and the Cook’s distances that measure the influence of each observation on the estimation of the model parameters are present. Cook’s distance values greater than 1 are suspect and indicate the presence of a possible outlier or poor model.

**Figure 3:**
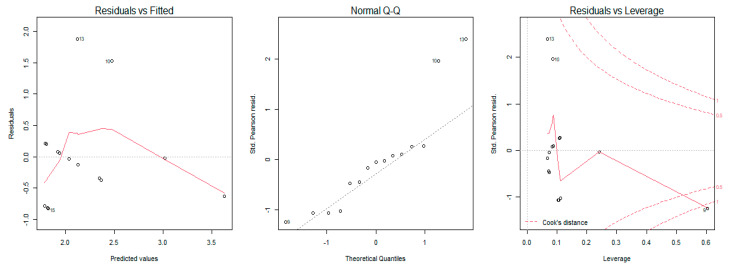
Graphical analysis of residuals for the MRC and sEMG model for Triceps Brachii muscle. The plot on the left shows the residual errors versus their estimated values; the QQ-plot in the center shows the distributive normality of the residuals; the plot on the right identifies any influential data points on the model.

**Figure 4:**
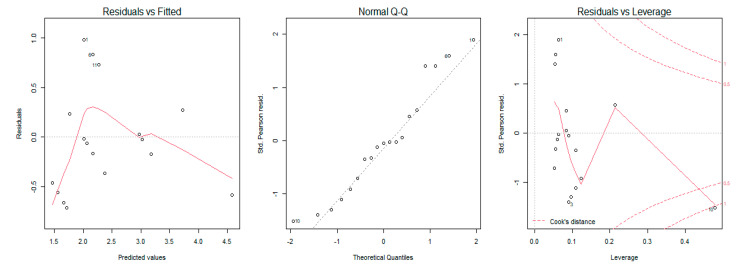
Graphical analysis of residuals for the MRC and Dynamometry model for Biceps Brachii muscle. The plot on the left shows the residual errors versus their estimated values; the QQ-plot in the center shows the distributive normality of the residuals; the plot on the right identifies any influential data points on the model.

**Figure 5:**
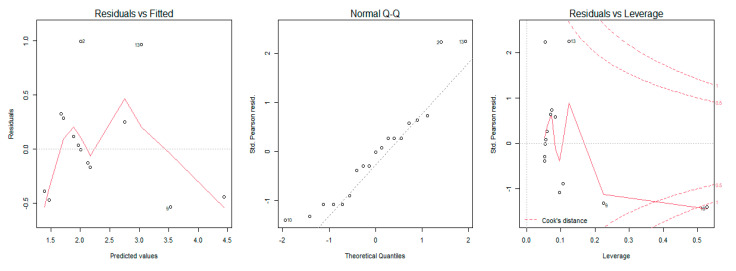
Graphical analysis of residuals for the MRC and Dynamometry model for Triceps Brachii muscle. The plot on the left shows the residual errors versus their estimated values; the QQ-plot in the center shows the distributive normality of the residuals; the plot on the right identifies any influential data points on the model.

A generalized regression model was used to study the relationship between the MRC scale scores, sEMG amplitude, and dynamometry measures of maximal elbow flexion and extension torque. The regression model showed that an increase of muscular strength by one point on the MRC scale was related to an increase of 59 mV (millivolts) of biceps brachii sEMG amplitude (% of explained variance = 0.50, Figure 2) and 83 mV for the triceps brachii sEMG amplitude (% of explained variance = 0.31, Figure 3). Moreover, the results revealed that a one-point increase on the MRC scale evaluation corresponded to an increase of 20 N (newtons) of elbow flexion torque measured with dynamometry (% of explained variance = 0.70, Figure 4) and 24 N of elbow extension torque (% of explained variance = 0.76, Figure 5).

**Figure 6:**
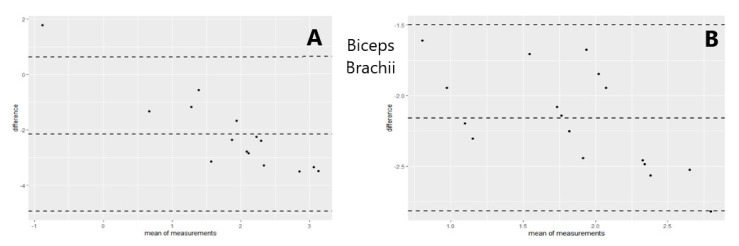
Differences between MRC scale and sEMG (**A**) as well as MRC and Dynamometry (**B**) for Biceps Brachii muscle versus the mean of the two measurements. The central line represents the mean difference (bias A = −2.16; B = −2.16), while the top and bottom lines represent the relative 95% CI (A = −4.93; 0.62) (B = −2.82; −1.50). The agreement between the measures is good when the differences are randomly distributed and fall within the 95% CI. The Bland–Altman plots highlight the differences of measurements performed with the two instruments. When the points (representing the observations) are scattered within the CI, the instruments can be used interchangeably. This mean that there are no significant differences between the measurements obtained from both instruments.

**Figure 7:**
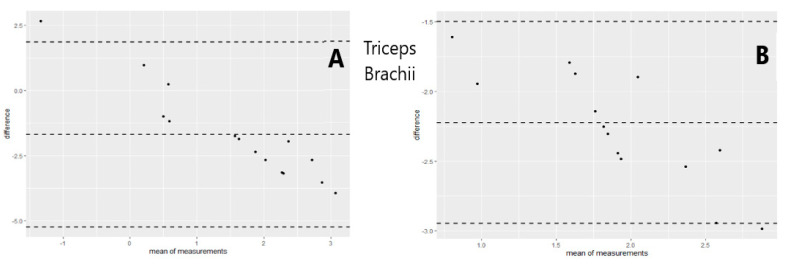
Differences between MRC scale and sEMG (**A**) as well as MRC and Dynamometry (**B**) for Triceps Brachii muscle versus the mean of the two measurements. The central line represents the mean difference (bias A = −1.60; B = −2.22), while the top and bottom lines represent the relative 95% CI (A = −5.23; 1.85) (B = −2.95; −1.49). The Bland–Altman plots highlight the differences of measurements performed with the two instruments. When the points (representing the observations) are scattered within the CI, the instruments can be used interchangeably. This mean that there are no significant differences between the measurements obtained from both instruments.

The goodness of fit of the first and second models showed normal distribution of residuals, whereas the goodness of fit of the third and fourth models showed non-normal distribution (Table 3). The QQ Plot (Figure 2 and Figure 3) and the Shapiro–Wilk normality test, performed on the two MRC biceps brachii models, confirmed the hypothesis of normality, both for the residuals of the model estimated with sEMG (W = 0.96 *p* = 0.77) and for those estimated with dynamometry (W = 0.93 *p* = 0.17). On the other hand, the goodness of fit carried out on the models of MRC triceps brachii did not have a normal distribution of residuals for the model estimated with sEMG (W = 0.83 *p* = 0.009) or for the model estimated with dynamometry (W = 0.89 *p* = 0.03). In all the Residuals versus Fitted graphs (Figure 2, Figure 3, Figure 4 and Figure 5), the points on the graph were random and did not show any evident pattern, a sign that there was no residual systematic dependence not identified from the estimated model. Some Residuals versus Leverage plots (Figure 3, Figure 4 and Figure 5) highlighted the presence of observations that could be considered outliers (they exceed the dotted line of Cook’s distance) and had an influence on the model estimation as the high Leverage values suggested. Furthermore, the Bland–Altman plots highlight the presence of a linear decreasing dependence, thus excluding the presence of significant bias. These results showed that applied assessment tools (i.e., MRC, Dynamometer, EMG) were comparable; however, differences were also present (Figure 6 and Figure 7).

## 4. Discussion

The results of this study showed that for the post-intervention data, muscular strength measured by the MRC scale was correlated to both the amplitude of muscle activity measured by sEMG, as well as measures of maximal voluntary torque assessed with dynamometry. Thus, the clinical measure of muscle strength in patients with hemiparesis increased in accordance with the changes observed in sEMG and dynamometry measures. We also observed that the MRC scale score and dynamometry measures were correlated when examining the pre-intervention data, but a similar correlation was not present between the MRC scale score and sEMG measures pre-intervention. This is likely due to the severe weaknesses of the tested muscles pre-intervention, which mostly did not present an active voluntary contraction.

In a study by Deroide et al., which investigated patients with neuropathic conditions, the EMG at baseline and during a MVC were weakly but significantly correlated to the MRC score [44]. Considering that a possible association between EMG and muscular force may help in the assessment of various aspects of muscle physiology, the addition of sEMG measurements for the evaluation of changes in muscle function following an intervention could support the interpretation of the MRC scale scores. This may also help to overcome some of the limitations of the MRC scale; for example, the original MRC scale does not include ROM. Consider an example of a person in the acute phase after stroke who can flex his/herelbow to 30° and after 1month of rehabilitation can flex to 70°. An improvement of 40° of flexion ROM is likely to be functionally significant; however, in both assessments (i.e., baseline and after rehabilitation), a grade 2 in the MRC scale will likely be obtained. Indeed, other authors have suggested that the MRC grading system should not be the sole outcome evaluation for elbow flexion strength, and quantitative measurements, such as using a dynamometer, should be included for outcome comparisons [45]. The results of our study suggest that inter-instrumental variation in muscle strength assessment can partly supplement the MRC scale outcome.

Direct measurement of muscle force using sEMG is not possible, and, although some studies have reported a linear relationship between force and sEMG amplitude [46], several others suggest a non-linear relationship [47]. In the current study, the regression model showed a linear relationship between the MRC scale score and sEMG amplitude as well as between the MRC scale score and dynamometry measures of elbow extension/flexion torque for triceps brachii and biceps brachii.The score on the MRC scale linearly increased with the amplitude observed during the sEMG acquisition and dynamometry assessment. This relationship supports the comparative outcome between the MRC scale and an instrumented assessment of muscle activity/torque during maximum voluntary contractions. Our findings provide new insights into the relationship between the measurements described above applied to plegic muscles resulting from central nervous system injury. This relationship does not explain exactly how much the muscle has recovered, but, from a clinical perspective, it can confirm the appropriateness of the interpretation of the applied MRC test. Despite the wide use of the MRC scale for strength assessment, this tool has been reported as not sufficiently sensitive and with limited accuracy to detect changes. Our results suggest that sEMG can be implemented for accurate assessment of post-stroke individuals when muscular force is evaluated. Thus, this may offer a more precise prediction of functional capabilities in patients with upper limb hemiparesis. The introduction of sEMG assessment can more easily detect and confirm muscle activity and/or residual force. This can be also helpful as a potential predictor of muscle force recovery. Collectively, our findings support the use of the MRC scale to evaluate changes in muscle strength and activity of the biceps and triceps brachii following rehabilitation in patients with severe hemiparesis.

There are some methodological considerations to note when interpreting the findings of this study. Firstly, we enrolled post-stroke patients with severe upper limb hemiparesis and, consequently, the presence of muscular fatigue and hypo-tone introduced non-linear distortions to the force–sEMG relationship, which may have limited this study [48,49]. A further consideration is that the catchment area of the electrode didnot extend sufficiently to detect the signal generated across the entire muscle volume.

Considering that the inter-rater reproducibility of the MRC scale had several limitations [50], future studies should also consider the correlation between MRC scores and instrumental assessments when data are collected from more than one assessor. Moreover, the residuals did not have a normal distribution for the triceps brachii and this may have been due to the small sample size of this study. Therefore, analysis of a larger sample and strength assessment of several muscle groups with both sEMG and dynamometry would provide a better understanding of the relationship between different methods of strength assessment and functional tasks. Another limitation is that we did not consider the potential effects of agonist–antagonist activation, which could have influenced the measures.

## 5. Conclusions

Variation in scores on the MRC scale was associated with variation in electromyographic activity as well as elbow torque measured with dynamometry. The findings of this study can be used to ensure more precise clinical assessments of patients with stroke.

## Figures and Tables

**Table 1:** Pre- and post-intervention values.

Clinical Parameters	Before Mean ± SD (95% CI)	After Mean ± SD (95% CI)
**MRC** (**No**) (biceps brachii)	0.42 ± 0.51 (0.18–0.67)	2.37 ± 0.96 (1.91–2.83)
**MRC** (**No**) (triceps brachii)	0.21 ± 0.42 (0.01–0.41)	2.16 ± 0.90 (1.73–2.59)
**Dynamometry** (**N**) (biceps brachii)	4.11 ± 6.04 (1.19–7.02)	23.00 ± 15.89 (15.34–30.66)
**Dynamometry** (**N**) (triceps brachii)	2.05 ± 5.45 (−0.58–4.68)	23.68 ± 18.93 (14.56–2.81)
**sEMG** (**mV**) (biceps brachii)	7.15 ± 8.89 (2.42–11.88)	40.04 ± 41.43 (17.09–62.98)
**sEMG** (**mV**) (triceps brachii)	2.04 ± 2.4 (0.71–3.37)	34.5 ± 43.16 (10.59–58.41)

Values are expressed as mean ± standard deviation (SD); sEMG, surface electromyography; MRC, Medical Research Council scale; No, points; N, newtons; mV, millivolts.

**Table 2:** Correlation between the MRC scale score and sEMG amplitude and Dynamometry measures.

Clinical Parameters	Before		After		∆	
	ρ	*p-*Value	ρ	*p-*Value	ρ	*p-*Value
**sEMG** (Biceps Brachii)	0.342 ^A^	0.1953	0.601 ^A^	0.0177 *	0.453	0.0898
**Dynamometry** (Biceps Brachii)	0.954 ^A^	0.0000 *	0.867 ^A^	0.0001 *	0.795	0.0000 *
**sEMG** (Triceps Brachii)	0.178 ^B^	0.5267	0.717 ^B^	0.0026 *	0.677	0.0079 *
**Dynamometry** (Triceps Brachii)	0.749 ^B^	0.0002 *	0.873 ^B^	0.0001 *	0.795	0.0000 *

ρ, correlation coefficient; *, *p*-value < 0.05; sEMG, surface electromyography; MRC, medical research council scale; Spearman’s rank correlation Test; A, MRC biceps brachii; B, MRC triceps brachii.

**Table 3:** Relationship between the MRC scale scores, sEMG amplitude, and Dynamometry.

Regression Model	% Variance Explained	*p-*Value of Residuals
**MRC** (biceps brachii) = 0.017 · **sEMG** (biceps brachii)	0.50	*p* = 0.766
**MRC** (biceps brachii) = 0.050 · **Dynamometry** (biceps brachii)	0.70	*p* = 0.165
**MRC** (triceps brachii) = 0.012 · **sEMG** (triceps brachii)	0.31	*p* = 0.009 *
**MRC** (triceps brachii) = 0.041 · **Dynamometry** (triceps brachii)	0.76	*p* = 0.033 *

The outcomes are displayed with equation of the regression models. The model was estimated on post-intervention data. The Normality test was applied on model’s residuals and significance was established at *p* < 0.05 *.

## Data Availability

The data presented in this study are available on request from the corresponding author.
